# Supplementary material for: The interactome of intact mitochondria by cross-linking mass spectrometry provides evidence for coexisting respiratory supercomplexes
Source: Mol Cell Proteomics. 2017 Dec 8;17(2):216–32. doi: 10.1074/mcp.RA117.000470 (PMC5795388; doi:10.1074/mcp.RA117.000470)
Supplement: Supplemental Data [file supp_RA117.000470_133922_0_supp_23978_fzffwf.zip › spectra_annotation/mito_spectra_annotation/228-1-4-1-4-1.pdf]

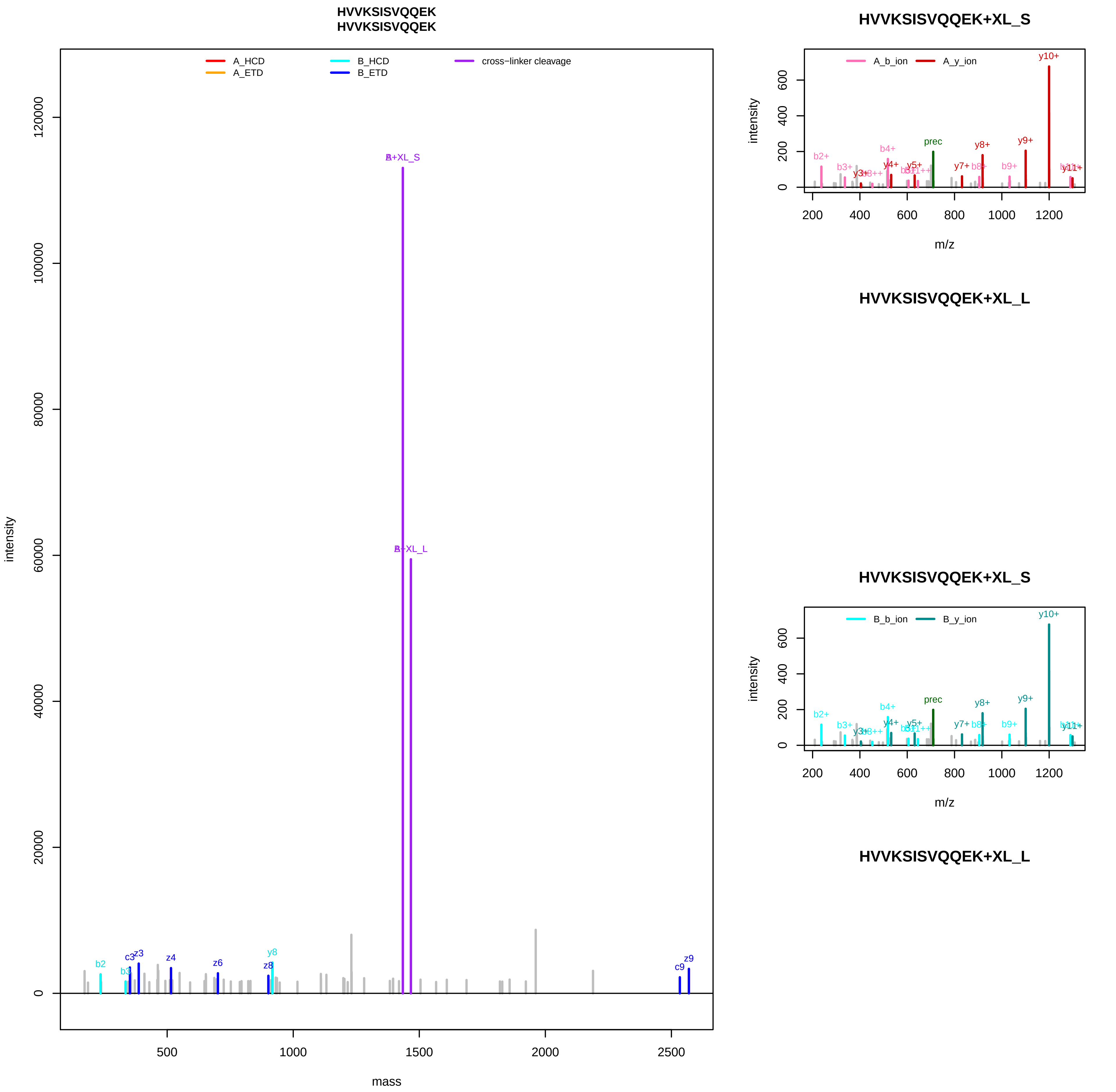

HVVKSISVQQEK
HVVKSISVQQEK
A_HCD
A_ETD
B_HCD
B_ETD
cross−linker cleavage
B+XL_S
B+XL_L
b2
b3
c3
z3
z4
z6
z8
y8
c9
z9
intensity
mass
0
20000
40000
60000
80000
100000
120000
500
1000
1500
2000
2500
HVVKSISVQQEK+XL_S
A_b_ion
A_y_ion
prec
b2+
b3+
b4+
y3+
y4+
y5+
y7+
y8+
y9+
y10+
b8+
b9+
y11+
intensity
m/z
0
200
400
600
800
1000
1200
HVVKSISVQQEK+XL_L
HVVKSISVQQEK+XL_S
B_b_ion
B_y_ion
prec
b2+
b3+
b4+
y3+
y4+
y5+
y7+
y8+
y9+
y10+
b8+
b9+
y11+
intensity
m/z
HVVKSISVQQEK+XL_L
